# Conserved sequences of BART and BHRF regions encoding viral microRNAs in Epstein-Barr virus-associated lymphoma

**DOI:** 10.1186/s13104-017-2603-z

**Published:** 2017-07-14

**Authors:** Keishin Sunagawa, Tsunekazu Hishima, Hitomi Fukumoto, Hideki Hasegawa, Harutaka Katano

**Affiliations:** 1 Department of Pathology, National Institute of Infectious Diseases, 1-23-1 Toyama, Shinjuku-ku, Tokyo, 162-8640 Japan; 2 Department of Pathology, Showa University School of Medicine, 1-5-8 Hatanodai, Shinagawa-ku, Tokyo, 142-8555 Japan; 3 Department of Pathology, Tokyo Metropolitan Komagome Hospital, 3-18-22 Honkomagome, Bunkyo-ku, Tokyo, 113-8677 Japan

**Keywords:** Epstein-Barr virus, miRNA, Lymphoma, PCR, Sequence

## Abstract

**Objective:**

Epstein-Barr virus (EBV) encodes at least 25 pri-microRNAs (miRNAs) in two regions of its DNA genome, BART and BHRF. B95-8, an EBV reference strain, has a deletion in the BART region. However, no information is available on the deletions or mutations in the BART and BHRF regions in clinical samples of EBV-associated lymphoma.

**Results:**

Nine DNA fragments encoding miR-BARTs and two coding miR-BHRF1s were amplified by PCR from DNA samples extracted from 16 cases of EBV-associated lymphoma. All the PCR products were sequenced directly. DNA fragments encoding miR-BARTs and miR-BHRF1-1 were successfully amplified from all samples. An adenine-to-guanine mutation in the DNA fragment encoding miR-BART2-3p was detected in four of the 16 cases, and a cytosine-to-thymidine mutation in the DNA fragment encoding miR-BART11-3p was detected in one of the 16 samples. These mutations were not associated with any histological categories of lymphoma. In conclusion, mutations were rarely observed in the DNA encoding viral miRNAs in cases of lymphoma. This suggests that the DNA sequences of EBV-encoded miR-BARTs and miR-BHRF1-1 are conserved in EBV-associated lymphoma.

## Introduction

Epstein-Barr virus (EBV) is a common herpesvirus that infects more than 90% of all adults throughout the world [1]. Primary EBV infections in adults cause infectious mononucleosis [2]. EBV is also associated with several types of lymphoma and cancer. According to a recent classification of lymphoma, EBV is associated with certain cases of Burkitt lymphoma, diffuse large-B-cell lymphoma (DLBCL) in immunodeficient patients, EBV-positive DLBCL of the elderly (ELD), DLBCL consistent with methotrexate (MTX)-associated lymphoproliferative disorder, NK/T-cell lymphoma (NKT), plasmablastic lymphoma, Hodgkin lymphoma, etc. [3]. Recently, it has been demonstrated that EBV encodes at least 25 pre-microRNAs (miRNAs) in its genome and that 44 mature miRNAs are registered in miRBase (http://www.mirbase.org/) from these pre-miRNAs [4, 5]. miRNAs are small RNAs (20–22 nucleotides) with various demonstrated biological functions in cells [6]. Representative functions of miRNAs include RNA silencing and the posttranscriptional regulation of gene expression by binding their target mRNAs [7]. The miRNAs of EBV are encoded in two primary transcripts, BHRF1 and BART. BHRF1 encodes miR-BHRF1-1, 1-2, and 1-3, which are expressed in EBV latency III infection [8]. BART encodes 22 miR-BARTs in three clusters [9]. Various functions of the EBV-encoded miRNAs have been reported. However, B95-8, an EBV reference strain, has a deletion in the BART DNA [10], although it displays full transformational activity in human B cells. In contrast, a study of insertion mutants of miR-BARTs in a recombinant EBV (B95-8) genome demonstrated that miR-BARTs promote cell-cycle progression and prevent the apoptosis of primary human B cells [11]. Another study showed that miR-BHRF1s accelerate B-cell expansion with low latent gene expression levels and reduce the viral antigenic load, which potentially facilitate the persistence of the virus in the infected host [12]. Therefore, the functions of the miR-BARTs and miR-BHRF1s in B-cell transformation differ.

A recent study of the full-genome sequences of EBV using next-generation sequencing demonstrated that BART and other regions were deleted in the full EBV genomes of some EBV-positive cell lines [13]. However, because the BART region contains a repetitive sequence, the next-generation sequencer did not correctly read the full sequence of BART. Therefore, there is no information about the deletions or mutations in the BART and BHRF regions in clinical samples of EBV-associated lymphoma.

In this study, DNA fragments encoding miR-BARTs and miR-BHRF1 were amplified by PCR from DNA samples extracted from EBV-positive lymphoma patients and were directly sequenced to determine the mutation, deletion, and/or insertion status of the regions encoding miRNAs.

## Main text

### Clinical specimens

Sixteen frozen samples of EBV-associated lymphoma were examined. All samples were obtained in biopsy or autopsy for pathological diagnosis. The samples included seven ELD, three AIDS-related DLBCLs (ARL), two NKTs, two low-grade B-cell lymphomas, one MTX-related lymphoma (MTX), and one plasmablastic lymphoma (PBL). All the patients were residents of Tokyo or prefectures neighboring Tokyo. The histological diagnosis of lymphoma was based on the 4th edition of the World Health Organization classification of lymphoma [3]. All cases were confirmed as positive for EBV infection with the in situ hybridization of EBV-encoded small RNAs (EBER). In situ hybridization was performed using EBER PNA Probe/Fluorescein and PNA-ISH Detection Kit (Dako, Glostrup, Denmark) according to the manufacturer’s instructions.

### PCR amplification and DNA sequencing

DNA was extracted from frozen samples from lymphoma patients, with the DNeasy Blood & Tissue Kit (Qiagen, Hilden, Germany), according to the manufacturer’s protocol. Fragments containing the all EBV-miRNA genes were amplified with PCR from the DNA samples using the primers listed in Table 1. PCR amplification was performed at 94 °C for 5 min (one cycle); 94 °C for 30 s, 55 °C for 30 s, and 72 °C for 30 s (35 cycles); and 72 °C for 10 min (one cycle) using the GeneAmp PCR System 9700 (Applied Biosystems, Foster City, CA, USA). The PCR products were purified with the QIAquick PCR Purification Kit (Qiagen), and then directly sequenced with an ABI 3130 Genetic Analyzer (Applied Biosystems) using the BigDye Terminator Ready Reaction Kit (Applied Biosystems), according to the manufacturer’s instructions.

**Table 1:** List of PCR primers

Set	Targets	Strand	Name of primer	Start	End	Sequence (5′-3′)	Size of PCR product
E1	miR-BARTs3,4,1	Forward	EBV139081F	139,081	139,100	tccctgtaaacacacaccac	440
		Reverse	EBV139520R	139,520	139,501	ttctacatcatgcctggttc	
E2	miR-BARTs15,5,16,17,6	Forward	EBV139501F	139,501	139,520	gaaccaggcatgatgtagaa	690
		Reverse	EBV140190R	140,190	140,171	tttagatctgtggttacatg	
E3	miR-BARTs21,18	Forward	EBV145451F	145,451	145,470	ttagatgttagctttgtgtt	590
		Reverse	EBV146040R	146,040	146,021	ggcccaaaccttcgcagcag	
E4	miR-BART7	Forward	EBV145911F	145,911	145,930	ttgttgccgttgaaagacgg	610
		Reverse	EBV146520R	146,520	146,501	tggccacactaaacacacaa	
E5	miR-BARTs8,9	Forward	EBV146701F	146,701	146,720	ttatttgggttacaagacct	350
		Reverse	EBV147050R	147,050	147,031	cacaatgaaacccaaagccc	
E6	miR-BARTs22, 10,11	Forward	EBV147131F	147,131	147,150	cggttgtcacaggtgctaga	500
		Reverse	EBV147630R	147,630	147,611	cgtgaaaggcactccagaat	
E7	miR-BARTs12,19,20	Forward	EBV147871F	147,871	147,890	acctaagacccgcccatcac	550
		Reverse	EBV148420R	148,420	148,401	ccaaaggacccgggatcacg	
E8	miR-BARTs13, 14	Forward	EBV148461F	148,461	148,480	catcttgacgttggaatgtc	360
		Reverse	EBV148820R	148,820	148,801	ctcctgggttggcgtttccg	
E9	miR-BART2	Forward	EBV152651F	152,651	152,670	gcagcaaaagaggaacttgc	350
		Reverse	EBV153000R	153,000	152,981	ggcaaagatccccagcggag	
E10	miR-BHRF1-1	Forward	EBV41581F	41,581	41,600	cctcaccatgacacactaag	260
		Reverse	EBV41840R	41,840	41,821	ccagatgcacccaacagccc	
E11	miR-BHRF1-2,3	Forward	EBV42991F	42,991	43,010	gggtgacacagtgcccatgc	330
		Reverse	EBV43320R	43,320	43,301	acactcacctcagttatttc	

Nucleotide numbering is based on GenBank KF373730

### Alignment analysis

Sequences were aligned with the Genetyx software (ver. 13, Genetyx, Tokyo, Japan). The reference sequences used were EBV strains AG876 (GenBank accession no. NC_009334), Akata (KC207813), B95-8 (V01555), HL01 (LN824226), HL02 (LN827546), Jijoye (LN827800), L591 (LN827523), Makaku (LN827551), Mutu (KC207814), Raji (KF717093), sLCL-IS1.20 (LN827576), and Wewaki1 (LN827544).

## Results

**Fig. 1:**
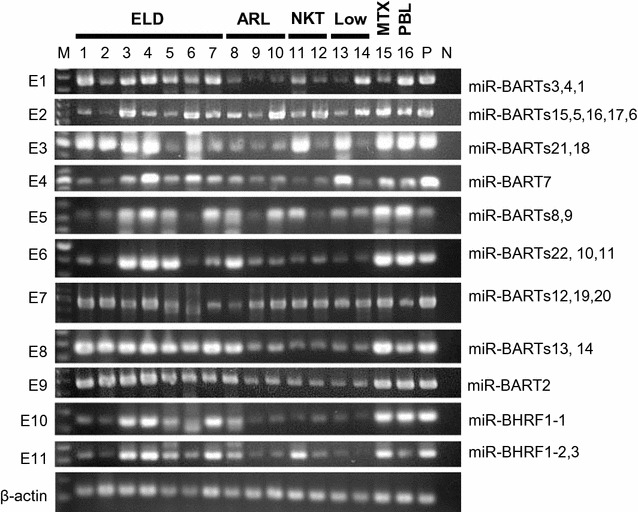
PCR products from EBV in clinical samples of lymphoma. The PCR products were separated electrophoretically in 2% agarose gel and stained with ethidium bromide. *M* 100-bp ladder molecular weight markers; samples *1–16*, *P* positive control (Raji, an EBV-positive Burkitt lymphoma cell line), and *N* no-DNA negative control

**Fig. 2:**
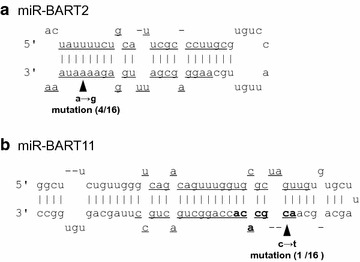
Mutations in pri-miR-BART2 and BART11. Structures of pri-miR-BART2 (**a**) and pri-miR-BART11 (**b**) are shown. *Black triangles* indicate single-nucleotide mutations. Mature miRNAs are indicated with *underlining*. Seed region of miR-BART11-3p is shown in *bold letters*

Nine DNA fragments encoding miR-BARTs and two encoding miR-BHRF1 were successfully amplified with PCR from the DNA samples from all 16 patients with EBV-positive lymphoma (Fig. 1). Direct sequencing of the PCR products revealed only two mutations in the DNA fragments encoding pre-miRNAs relative to the reference sequences. An adenine-to-guanine mutation in the DNA fragment encoding miR-BART2-3p was detected in four of 16 cases (25%; Fig. 2a), which included two ELD, one T-cell lymphoma, and one low-grade B-cell lymphoma. A cytosine-to-thymidine mutation was found in a DNA fragment encoding miR-BART11-3p in one (ELD) of the 16 cases (6.25%). The ELD case with a cytosine-to-thymidine mutation in miR-BART11-3p DNA did not have an adenine-to-guanine mutation in the DNA fragment encoding miR-BART2-3p. The mutation in miR-BART11-3p was in the previously reported seed region of the miRNA [14] (Fig. 2b). There were no single-nucleotide polymorphisms, mutations, insertions, or deletions in any of the DNA fragments encoding other pre-miRNAs, including pri-miR-BHRF1s, suggesting that the miRNA sequences are strongly conserved in cases of lymphoma.

## Discussion

The two mutations in the DNA fragment encoding miR-BARTs found in this study were not associated with any histological features of the lymphoma. In a previous study, miR-BART2-3p was not expressed in the serum samples of patients in the acute phase of infectious mononucleosis, whereas miR-BART2-5p was expressed abundantly [15]. Therefore, the mutation in miR-BART2-3p identified in the present study is unlikely to be associated with the lymphomagenesis of a specific category of lymphoma. However, miR-BART11-3p has been reported to be strongly expressed in infectious mononucleosis, gastric cancer, and nasopharyngeal carcinoma [14]. EBV-miR-BART11 plays a crucial role in the promotion of carcinogenesis in gastric cancer and nasopharyngeal carcinoma by inhibiting the tumor-suppressive effects of FOXP1 [14]. The mutation identified in the present study was in the seed region of miR-BART11-3p, suggesting that it affects the role of miR-BART11-3p.

In this study, the genetic conservation of the EBV DNA encoding miRNAs was demonstrated in patients with EBV-associated lymphoma, implying that deletions in BART are rare. The genome of Kaposi’s sarcoma-associated herpesvirus (KSHV) also encodes viral miRNAs [16], which reportedly have important functions in cell transformation and cell-cycle promotion [17, 18]. A study of clinical samples in which the DNA fragments encoding KSHV miRNAs were sequenced detected SNPs in the DNA fragments encoding miRNAs in multicentric Castleman disease and KSHV inflammatory cytokine syndrome, suggesting that sequence variations in KSHV miRNA are associated with these diseases [19]. However, no large deletion of DNA encoding an miRNA has been reported in KSHV-associated diseases. A recent study showed that a defect in the miR-BARTs region, as in B95-8, is uncommon among EBV-positive cell lines, although partial defects have been observed in two other cell lines [13]. However, the partial defects detected in that study were near the assembly gap region introduced by next-generation sequencing, suggesting that the defects were associated with the sequencing technique. The gap region was not PCR amplified in that study [13]. The lack of deletions detected in miR-BART in the present study suggests that these deletions do not occur or occur only rarely in clinical samples.

In conclusion, the sequences of the EBV genome that encode miR-BARTs and miR-BHRF1 are conserved in EBV-associated lymphoma. Further studies comparing cell lines and clinical samples are required to investigate the role of the BART deletions in EBV B95-8, and the association between the mutations in the DNA fragments encoding miR-BARTs and the pathogenesis of lymphoma.

## Limitations

The limitation of this study includes small numbers of samples and PCR amplification of limited regions. Therefore, the conclusion of this study may not be generalizable to all EBV-infected cases. Any functional analysis of the mutated miRNAs was not performed in this study. Despite these limitations, these findings suggest genomic conservation of EBV at least in a subset of lymphoma because there is no similar study.

## Abbreviations

AIDS: acquired immunodeficiency syndrome
ARL: AIDS-related diffuse large B cell lymphoma
DLBCL: diffuse large B-cell lymphoma
EBV: Epstein-Barr virus
ELD: EBV-positive DLBCL of the elderly
KSHV: Kaposi sarcoma-associated herpesvirus
LOW: low-grade B-cell lymphoma
miRNA: microRNA
MTX: methotrexate-related lymphoma
PBL: plasmablastic lymphoma
PCR: polymerase chain reaction
SNP: single nucleotide polymorphism
NKT: NK/T-cell lymphoma
